# Supplementary material for: Architectural control of metabolic plasticity in epithelial cancer cells
Source: Commun Biol. 2021 Mar 19;4:371. doi: 10.1038/s42003-021-01899-4 (PMC7979883; doi:10.1038/s42003-021-01899-4)
Supplement: Supplementary file 2 — Supplementary Information [file 42003_2021_1899_MOESM2_ESM.pdf]

Supplementary Figures

Architectural Control of Metabolic Plasticity in Epithelial Cancer Cells

Maia Al Masri[1,2], Karina Paliotti[1], Raymond Tran[3], Ruba Halaoui[1,2], Virginie Lelarge[1], Sudipa Chatterjee[1,2], Li-Ting Wang[1,2] , Christopher Moraes[1,3], Luke McCaffrey[1,2,4,5]

[1]Rosalind and Morris Goodman Cancer Research Centre, McGill University, Montreal, Canada
[2]Division of Experimental Medicine, McGill University, Montreal, Canada
[3]Department of Chemical Engineering, McGill University, Montreal, Canada
[4]Gerald Bronfman Department of Oncology, McGill University, Montreal, Canada
[5]Correspondence: luke.mccaffrey@mcgill.ca

Supplementary Figure S1

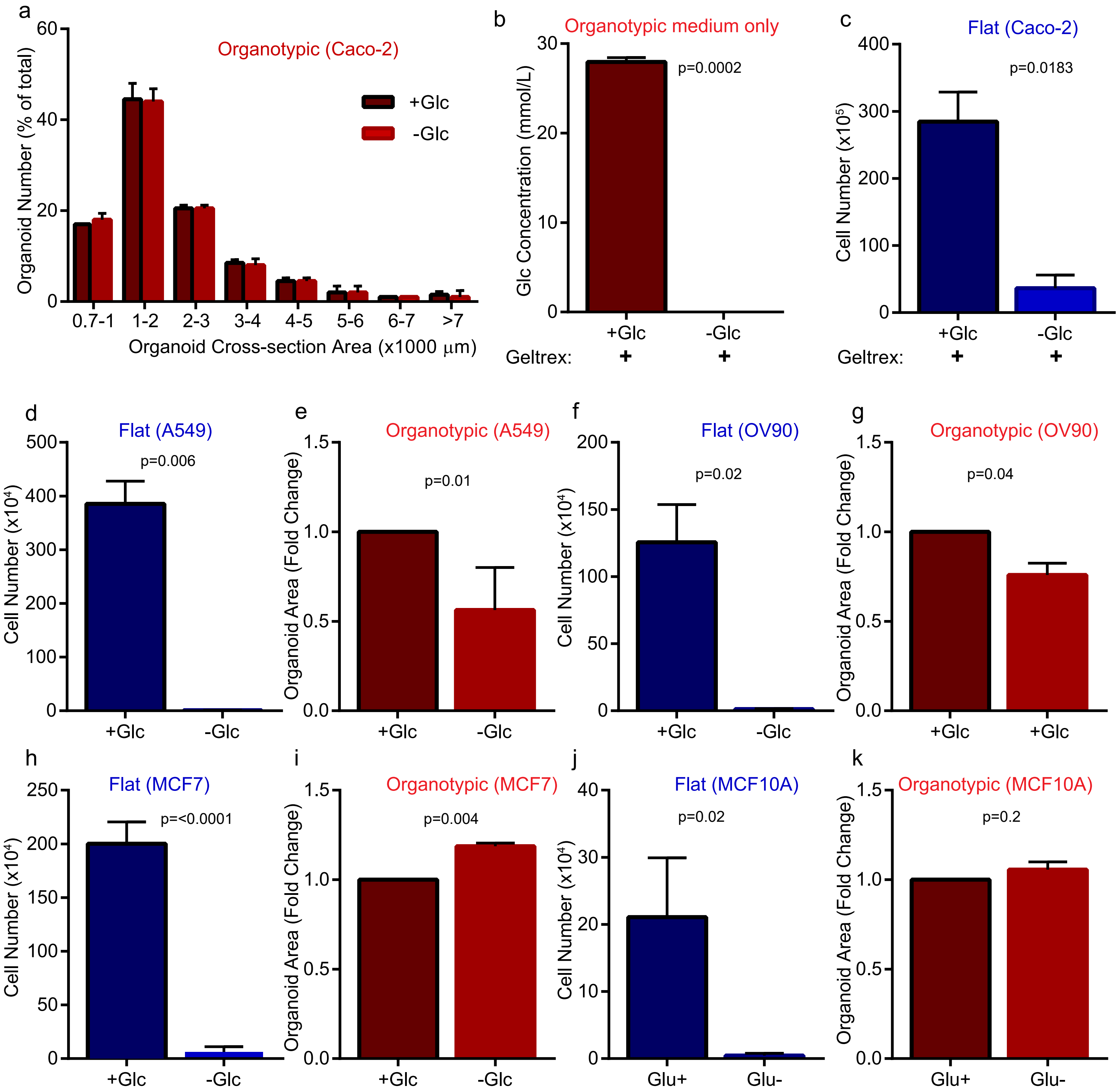

Supplementary Figure S1 - Epithelial architecture is associated with glucose-dependent growth
a) Histogram showing the distribution of Caco-2 organoid cross-section areas after 6-days of organotypic culture in the presence (+Glc) or absence (-Glc) of glucose (n=2677 (+Glc), n=2663 (-Glc)) (r=2 independent experimental replicates).

b) Bar chart showing the glucose concentration cell-free culture media containing Geltrex in the presence (+Glc) or absence (-Glc) of glucose (r=2 independent experimental replicates).

c) Bar chart showing Caco-2 cell number after 6 days of flat culture in media containing soluble basement membrane extract (BME) in the presence (+Glc) or absence (-Glc) of glucose (r=2 independent experimental replicates).

d) Bar chart showing A549 cell number after 6 days of flat culture in the presence (+Glc) or absence (-Glc) of glucose (r=2 independent experimental replicates).

e) Bar chart showing cross-section area of A549 clusters after 6 days of organotypic culture in the presence (+Glc) or absence (-Glc) of glucose (n=3048 (+Glc), n=3435(-Glc)) (r=4 independent experimental replicates).

f) Bar chart showing OV90 after 6 -days of flat culture in the presence (+Glc) or absence (-Glc) of glucose (r=2 independent experimental replicates).

g) Bar chart showing cross-section area of OV90 clusters after 6 days of organotypic culture in the presence (+Glc) or absence (-Glc) of glucose (n=859 (+Glc), n=592 (-Glc)) (r=2 independent experimental replicates).

h) Bar chart showing MCF7 cell number after 6 days of flat culture in the presence (+Glc) or absence (-Glc) of glucose (r=3 independent experimental replicates).

i) Bar chart showing cross-section area of MCF7 clusters after 6 days of organotypic culture in the presence (+Glc) or absence (-Glc) of glucose (n=680 (+Glc), n=944 (-Glc)) (r=2 independent experimental replicates).

j) Bar chart showing MCF10A cell number after 6 days of flat culture in the presence (+Glc) or absence (-Glc) of glucose (r=3 independent experimental replicates).

k) Bar chart showing cross-section area of MCF10A spheroids after 6 days of organotypic culture in the presence (+Glc) or absence (-Glc) of glucose (n=313 spheroids (+Glc), n=294 spheroids (-Glc)), (r=2 independent experimental replicates).

All error bars are standard deviation.

a

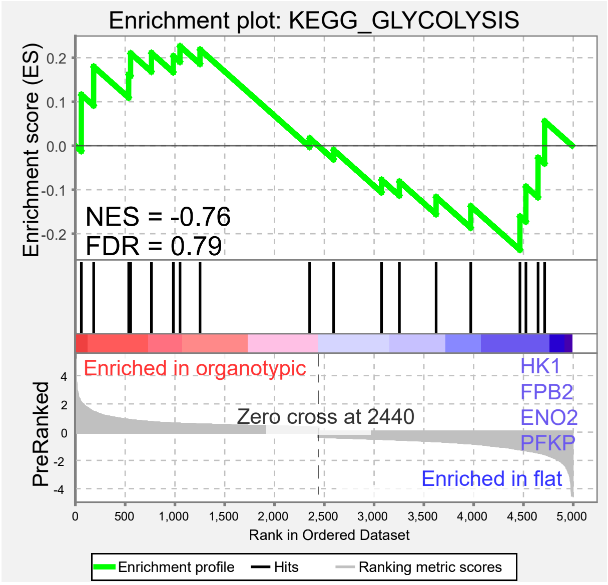

Enrichment plot: KEGG_GLYCOLYSIS
Enrichment score (ES)
NES = -0.76
FDR = 0.79
Enriched in organotypic
HK1
FPB2
ENO2
PFKP
Zero cross at 2440
Enriched in flat
PreRanked
Rank in Ordered Dataset
Enrichment profile
Hits
Ranking metric scores

b

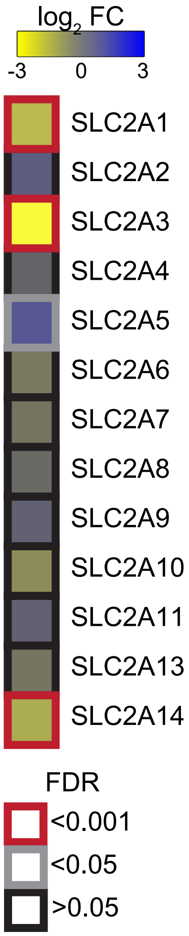

log_2 FC
-3
0
3
SLC2A1
SLC2A2
SLC2A3
SLC2A4
SLC2A5
SLC2A6
SLC2A7
SLC2A8
SLC2A9
SLC2A10
SLC2A11
SLC2A13
SLC2A14
FDR
<0.001
<0.05
>0.05

Supplementary Figure S2 – Enrichment of genes related to glycolysis

a) Gene Set Enrichment plot for KEGG_GLYCOLYSIS_GLUCONEOGENESIS gene set. Four genes in blue represente genes enriched (false discovery rate (FDR) < 0.01) in flat cultures.

b) Heatmap of gene expression for glucose transporters from RNA-Seq showing log2 fold change (FC) of organotypic compared to flat cultures. Outlines indicate the FDR for each gene. (Supplementary Table S1).

Supplementary Figure S3

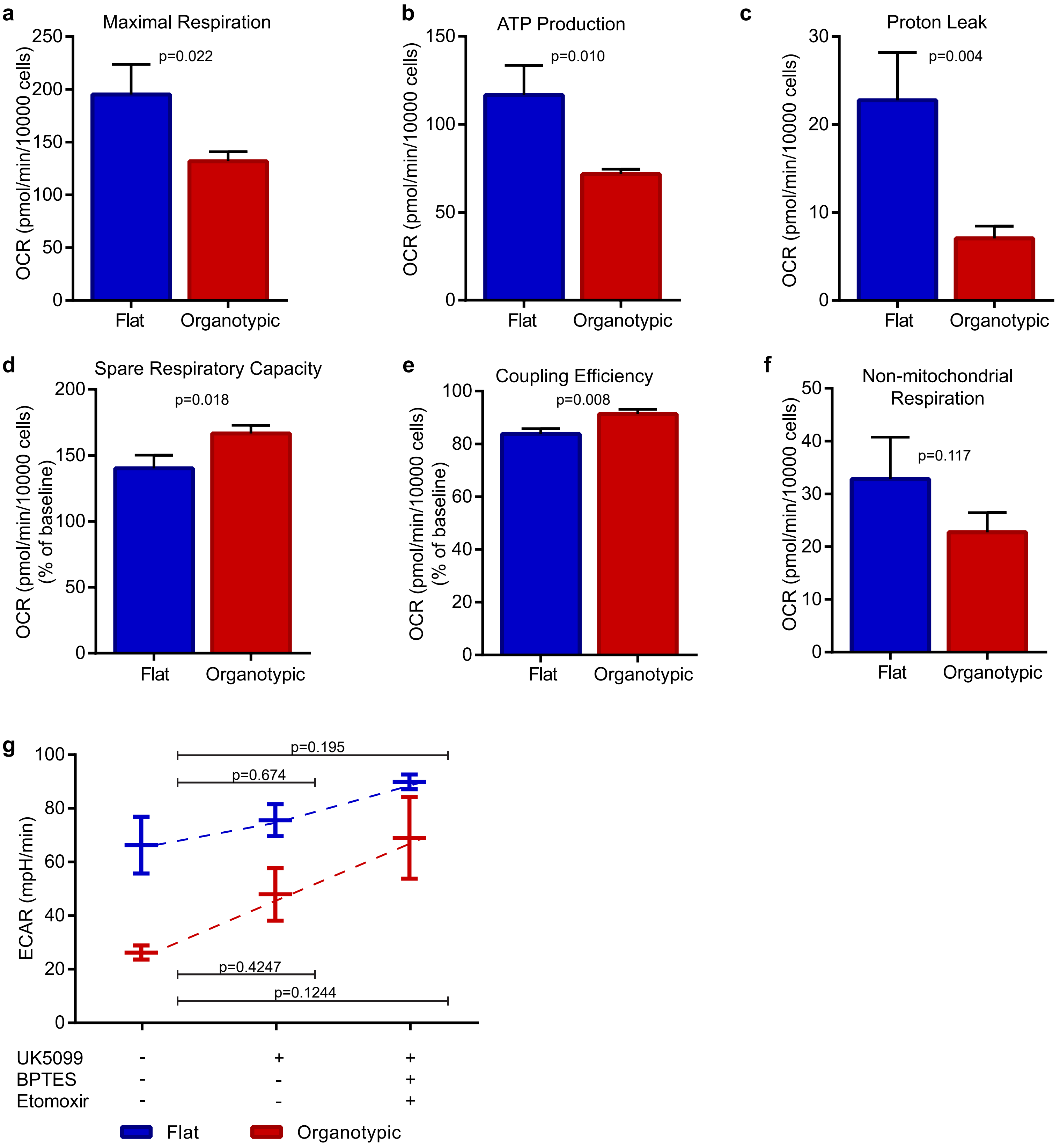

Supplementary Figure S3 – Epithelial architecture is associated with energetic stress responses
a-f) Bar charts showing maximal respiration **(a)**, ATP production **(b)**, proton leak **(c)**, spare respiratory capacity **(d)**, mitochondrial coupling efficiency **(e)**, and non-mitochondrial respiration **(f)** for flat and organotypic Caco-2 cultures (r=3 independent experimental replicates).

g) Waterfall chart showing ECARs at baseline and following sequential addition of UK5099 followed by BPTES and Etomoxir for flat and organotypic Caco-2 cultures (r=2 independent experimental replicates).
All error bars are standard deviation.

Supplementary Figure S4

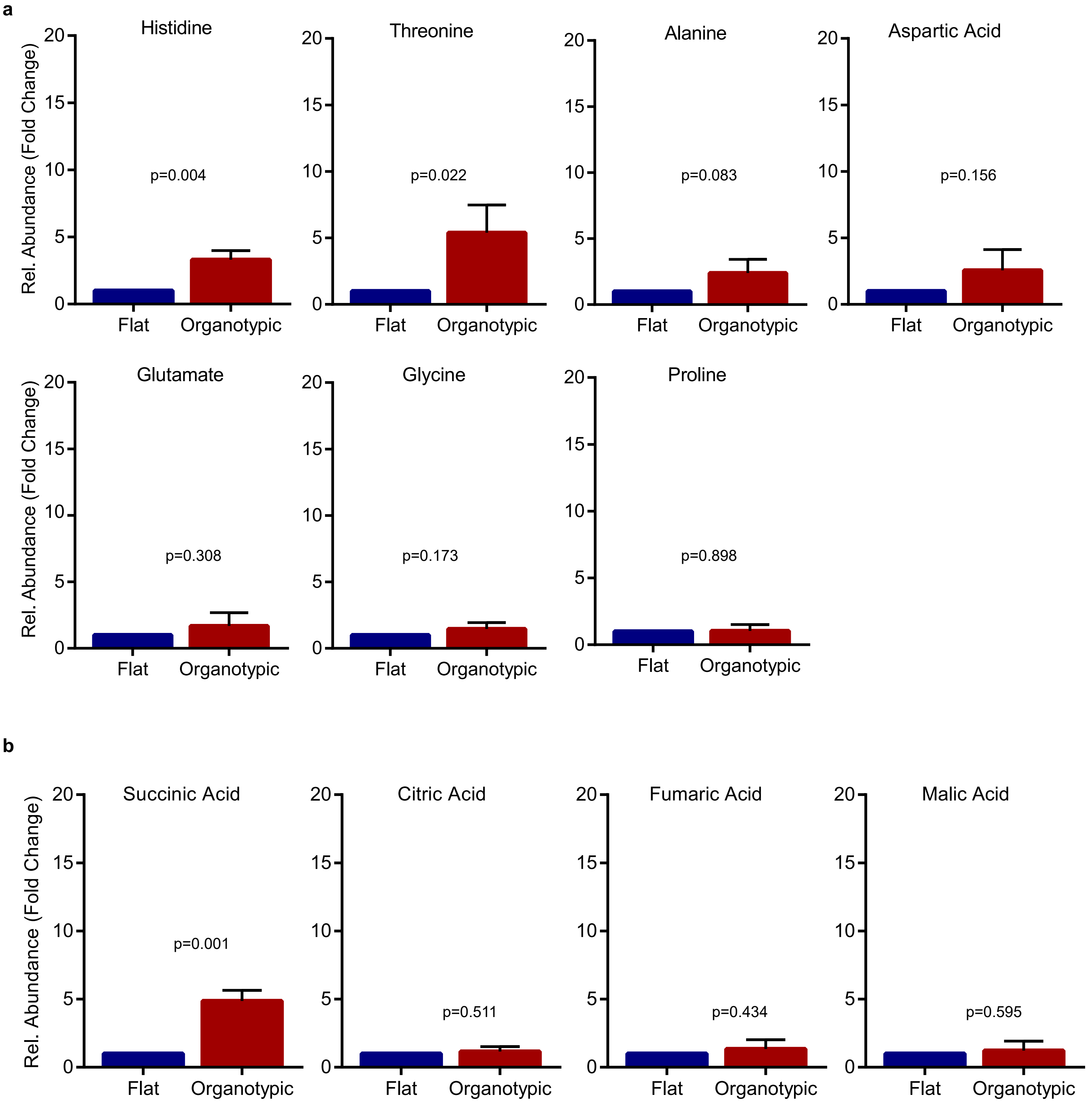

Supplementary Figure S4 – Amino acid metabolism is enriched in organotypic cultures

a) Bar graphs showing abundance of histidine, threonine, alanine, aspartic acid, glutamic acid, glycine, and proline from flat and organotypic Caco-2 cells. Cells were cultured for 5 days, then cellular extracts were analyzed by GC/MS. Data available in Supplementary Table S2. (n=5 sample replicates for each of r=3 independent experimental replicates).

b) Bar graphs showing normalized levels of succinic acid, citric acid, fumaric acid, and malic acid from flat and organotypic Caco-2 cells. (n=5 sample replicates for each of r=3 independent experimental replicates).

All error bars are standard deviation.

Supplementary Figure S5

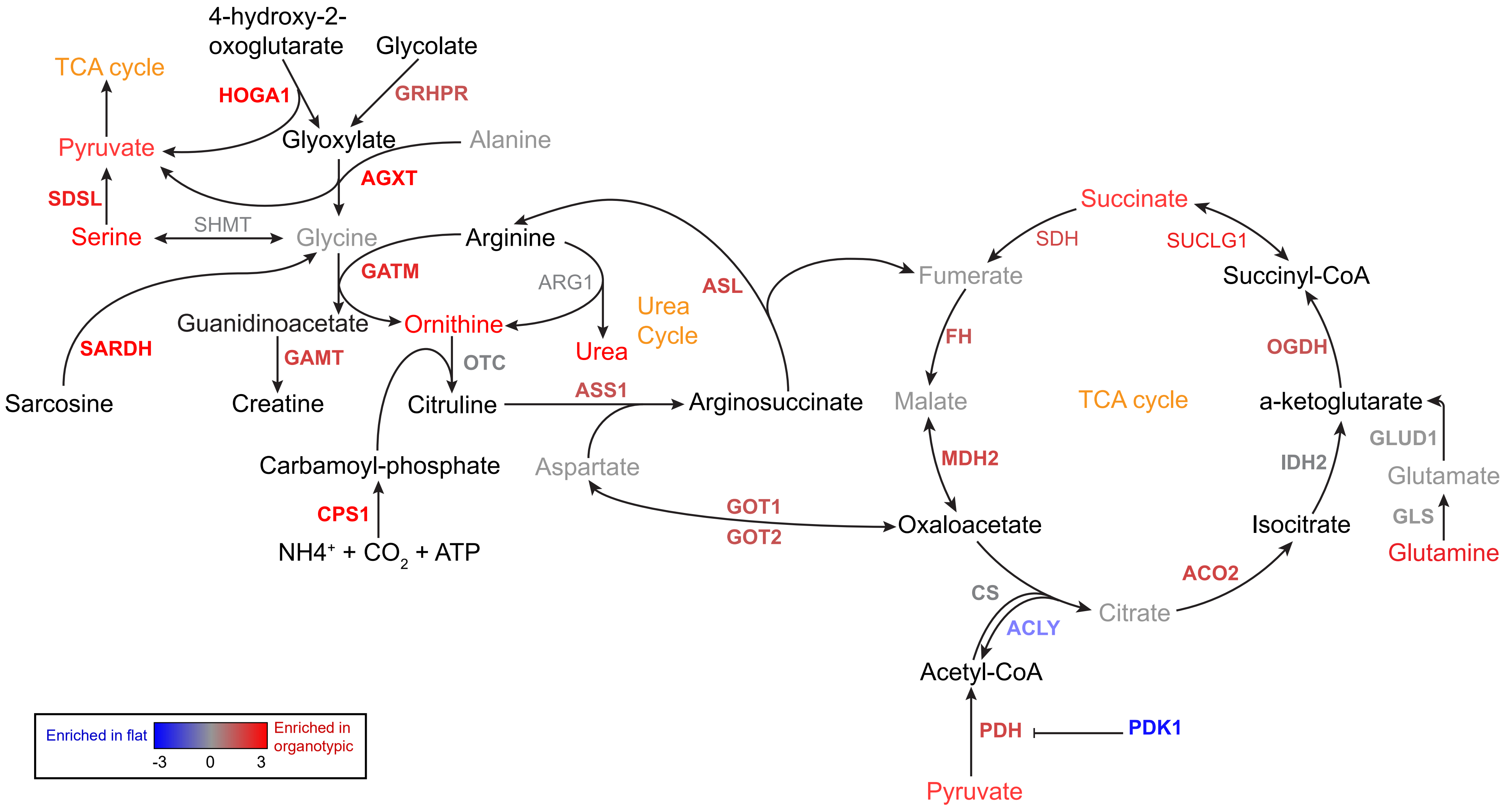

Supplementary Figure S5 – Differences in metabolic networks in flat and organotypic cultures

Metabolic map showing integrated gene-expression and GC/MS data for metabolic pathways enriched in organotypic Caco-2 cultures. Red indicates enrichment in organotypic cultures and blue indicates enrichment in flat cultures. Grey indicates no enrichment and black indicates the gene/metabolite was not measured.

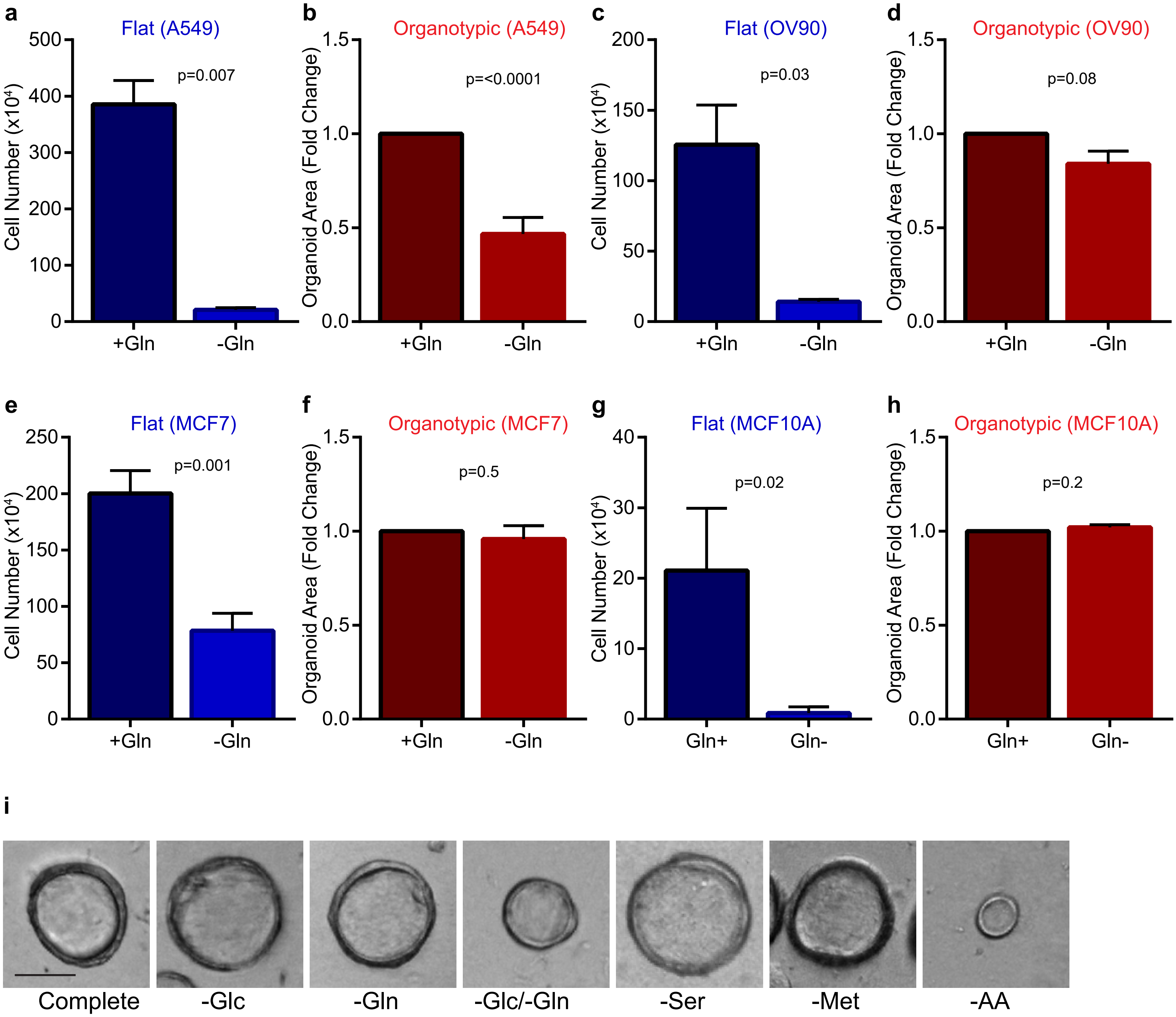
a
Flat (A549)
Cell Number (x10⁴)
p=0.007
+Gln
-Gln
b
Organotypic (A549)
Organoid Area (Fold Change)
p=<0.0001
+Gln
-Gln
c
Flat (OV90)
Cell Number (x10⁴)
p=0.03
+Gln
-Gln
d
Organotypic (OV90)
Organoid Area (Fold Change)
p=0.08
+Gln
-Gln
e
Flat (MCF7)
Cell Number (x10⁴)
p=0.001
+Gln
-Gln
f
Organotypic (MCF7)
Organoid Area (Fold Change)
p=0.5
+Gln
-Gln
g
Flat (MCF10A)
Cell Number (x10⁴)
p=0.02
Gln+
Gln-
h
Organotypic (MCF10A)
Organoid Area (Fold Change)
p=0.2
Gln+
Gln-
i
Complete
-Glc
-Gln
-Glc/-Gln
-Ser
-Met
-AA

Supplementary Figure S6 - Organotypic cells exhibit plasticity to Amino Acid nutrient stress
a) Bar chart showing A549 cell number after 6 days of flat culture in the presence (+Gln) or absence (-Gln) of glutamine (n=2 independent experimental replicates).

b) Bar chart showing cross-section area of A549 cell clusters after 6 days of organotypic culture in the presence (+Gln) or absence (-Gln) of glutamine (n=3048(+Gln), n=2872(-Gln)) (r=4 independent experimental replicates).

c) Bar chart showing OV90 cell number after 6 days of flat culture in the presence (+Gln) or absence (-Gln) of glutamine (n=2 independent experimental replicates).

d) Bar chart showing cross-section area of OV90 cell clusters after 6 days of organotypic culture in the presence (+Gln) or absence (-Gln) of glutamine (n=859(+Gln), n=821(-Gln)) (r=2 independent experimental replicates).

e) Bar chart showing MCF7 cell number after 6 days of flat culture in the presence (+Gln) or absence (-Gln) of glutamine (n=2 independent experimental replicates).

f) Bar chart showing cross-section area of MCF7 cell clusters after 6 days of organotypic culture in the presence (+Gln) or absence (-Gln) of glutamine (n=680(+Gln), n=829(-Gln)) (r=2 independent experimental replicates).

e) Bar chart showing MCF10A cell number after 6 days of flat culture in the presence (+Gln) or absence (-Gln) of glutamine (n=2 independent experimental replicates).

f) Bar chart showing cross-section area of MCF10A cell clusters after 6 days of organotypic culture in the presence (+Gln) or absence (-Gln) of glutamine (n=359 (+Gln), n=316 (-Gln)); (r=2 independent experimental replicates).

i) Representative phase contrast images from Caco-2 organotypic cultures showing architecture of cell cysts grown in complete medium, or medium free of glucose (-Glc), glutamine (-Gln), glucose and glutamine (-Glc/Gln), serine (-Ser), methionine (-Met), and amino acids (-AA).
All error bars are standard deviation.

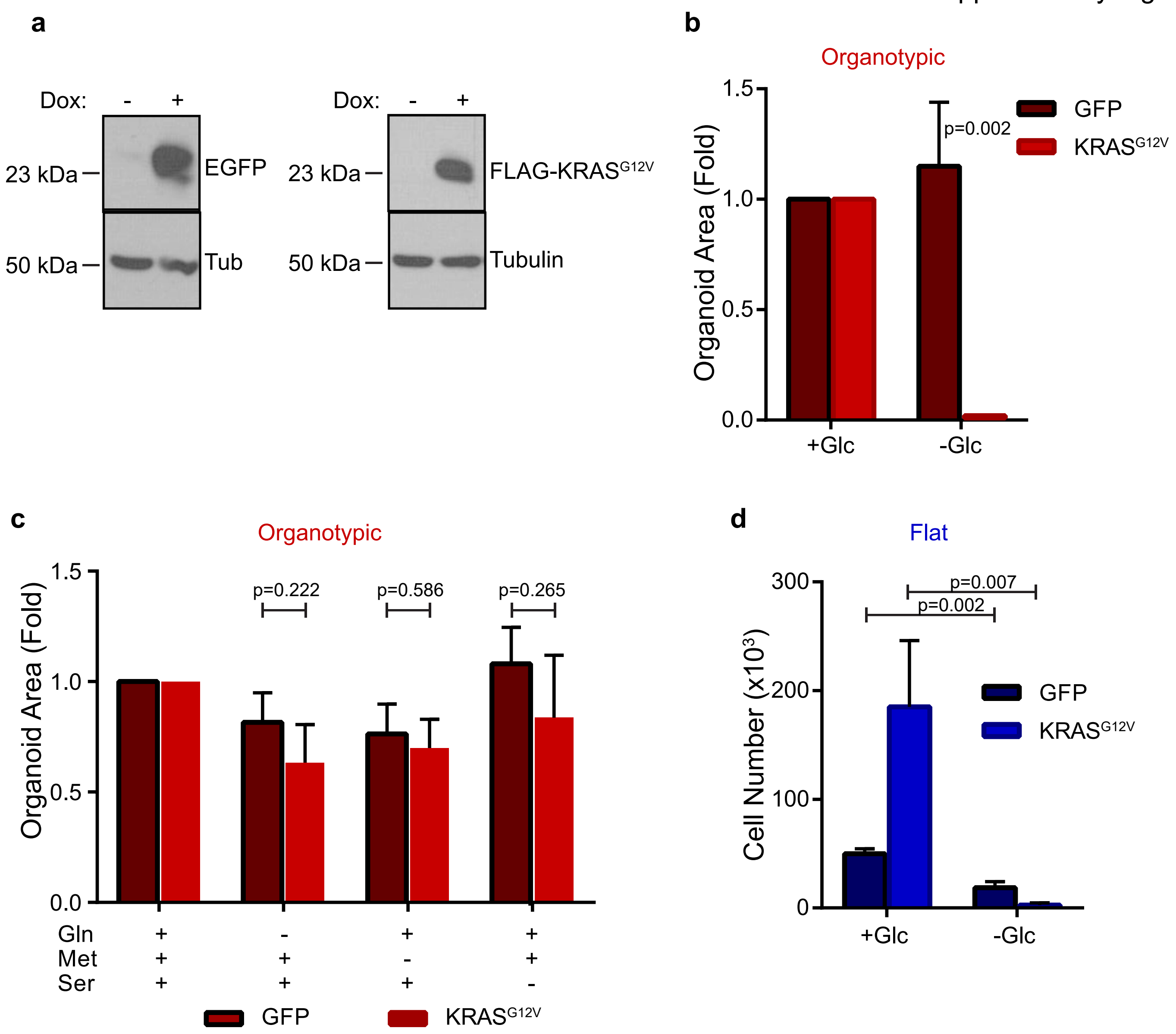
a
Dox: - +
23 kDa
EGFP
50 kDa
Tub
Dox: - +
23 kDa
FLAG-KRASG12V
50 kDa
Tubulin
b
Organotypic
Organoid Area (Fold)
1.5
1.0
0.5
0.0
p=0.002
GFP
KRASG12V
+Glc
-Glc
c
Organotypic
Organoid Area (Fold)
1.5
1.0
0.5
0.0
p=0.222
p=0.586
p=0.265
Gln + - + +
Met + + - +
Ser + + + -
GFP
KRASG12V
d
Flat
Cell Number (x103)
300
200
100
0
p=0.007
p=0.002
GFP
KRASG12V
+Glc
-Glc

Supplementary Figure S7 – Organotypic cultures have reduced metabolic plasticity with Ras-driven glucose addiction
a) Immunoblot showing doxycycline-inducible (+/- Dox) expression of GFP and FLAG-tagged $KRAS^{G12V}$ in Caco-2 cells using anti-GFP and anti-FLAG antibodies, respectively. Tubulin (Tub) was included as a loading control. Molecular weights (kDa) are indicated on the left.

b) Bar chart showing cross-section area of control (GFP) and $KRAS^{G12V}$-expressing organotypic Caco-2 cells in the presence (+Glc) or absence (-Glc) of glucose (GFP: n=888 (+Glc), n= 898 (-Glu); $KRAS^{G12V}$: n=975 (+Glc) n=0 (-Glc)); (r=3 independent experimental replicates).

c) Bar chart showing cross-section area of control (GFP) and $KRAS^{G12V}$-expressing organotypic cells in the presence (+) or absence (-) of glutamine (Gln), methionine (Met), or serine (Ser) (GFP: n=888 (+Gln, +Met, +Ser), n= 916 (-Gln), n= 585 (-Met), n=759 (-Ser); $KRAS^{G12V}$: n=975 (+Gln, +Met, +Ser) n=1106 (-Gln), n=1103 (-Met), n=999 (-Ser)); (r=3 independent experimental replicates).

d) Bar chart showing cell number for GFP and $KRAS^{G12V}$-expressing Caco-2 flat cells in the presence (+Glc) or absence (-Glc) of glucose); (r=3 independent experimental replicates).
All error bars are standard deviation.

Supplementary Figure S8

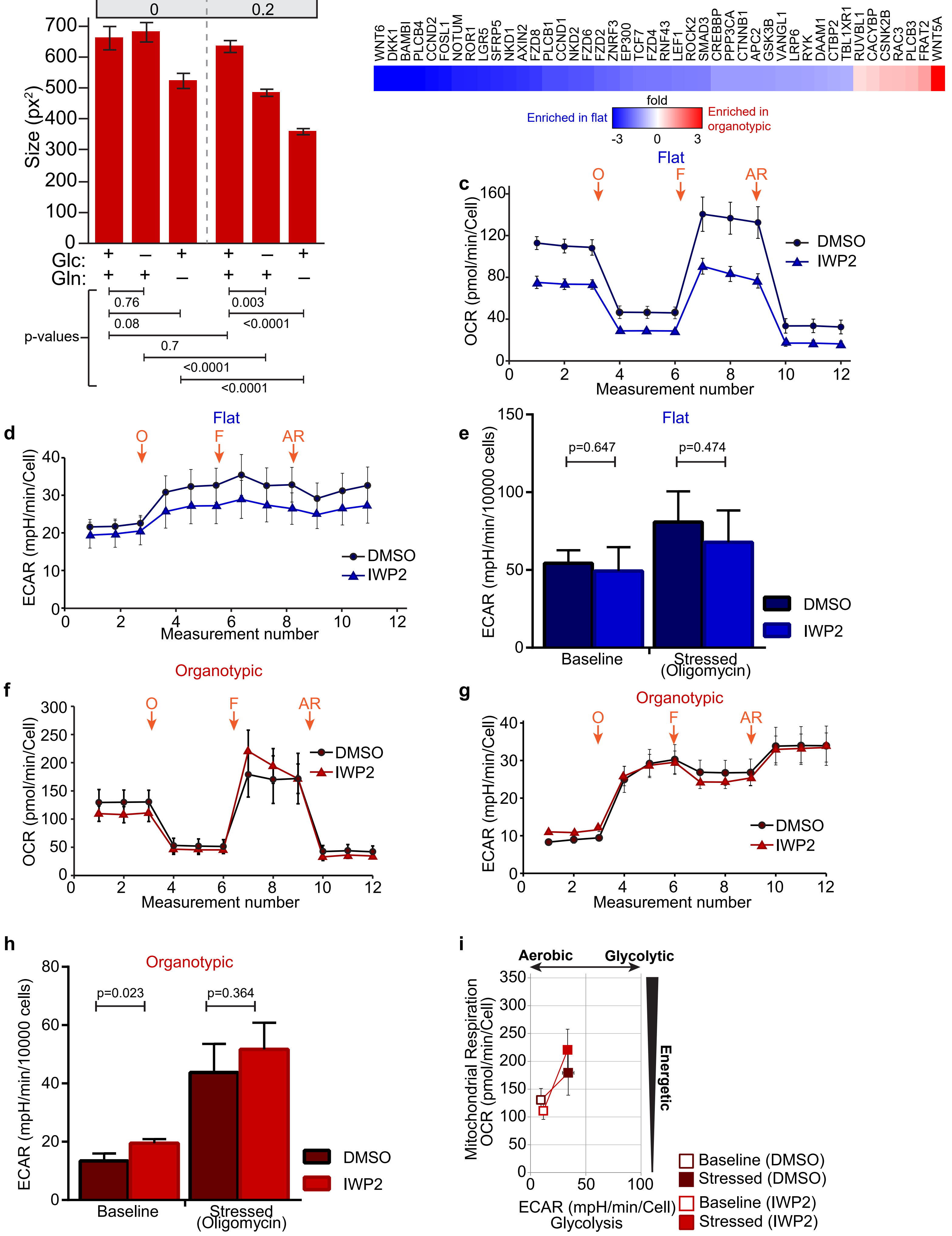

Supplementary Figure S8 – Mechanical regulation of metabolic plasticity correlates with Wnt signaling

a) Bar chart showing the sizes of Caco-2 spheroids in organotypic cultures cells in complete (Glc/Gln+), glucose-depleted (Glc-), or glutamine-depleted (Gln-) medium. Cells were cultured embedded in 50% basement membrane extract (BME) in the presence of 0 or 2% alginate for 5 days. For 0% alginate: n=319 (Glc+/Gln+), n=254 (Glc-), and n=127 (Gln-) spheroids were measured; for 0.2% alginate: n=234 (Glc+/Gln+), n=254 (Glc-), and n=127 (Gln-) (r=2 independent replicates).

b) Heat map showing enrichment of canonical Wnt-associated genes in flat cultures based on differential gene expression data (Supplementary Table S1). Wnt-associated genes are annotated from the KEGG_WNT_SIGNALING_PATHWAY gene set.

c) Representative line graph showing OCR measurements from control (DMSO) and IWP2-treated flat Caco-2 cultures in response to mitochondrial stressors (Oligomycin (O), FCCP (F), and Antimycin A with Rotenone (AR).

d) Representative line graph showing ECAR measurements from control (DMSO) and IWP2-treated flat Caco-2 cultures in response to mitochondrial stressors (Oligomycin (O), FCCP (F), and Antimycin A with Rotenone (AR).

e) Bar chart showing ECAR measurements from control (DMSO) and IWP2-treated flat Caco-2 cultures at baseline and following oligomycin-induced mitochondrial stress (r=3 independent experimental replicates).

f) Representative line graph showing OCR measurements from control (DMSO) and IWP2-treated organotypic Caco-2 cultures in response to mitochondrial stressors (Oligomycin (O), FCCP (F), and Antimycin A with Rotenone (AR).

g) Representative line graph showing ECAR measurements from control (DMSO) and IWP2-treated organotypic Caco-2 cultures in response to mitochondrial stressors (Oligomycin (O), FCCP (F), and Antimycin A with Rotenone (AR).

h) Bar chart showing ECAR measurements from control (DMSO) and IWP2-treated organotypic Caco-2 cultures at baseline and following oligomycin-induced mitochondrial stress (r=3 independent experimental replicates).

i) Plot showing the bioenergetic profile of control or IWP2-treated organotypic Caco-2 cultures at baseline activity (open symbols) and in response to mitochondrial oxidative phosphorylation uncoupler, FCCP (closed symbols) (r=3 independent experimental replicates).
