# Supplementary material for: Architectural control of metabolic plasticity in epithelial cancer cells
Source: Commun Biol. 2021 Mar 19;4:371. doi: 10.1038/s42003-021-01899-4 (PMC7979883; doi:10.1038/s42003-021-01899-4)
Supplement: Supplementary file 3 — Description of Additional Supplementary Files [file 42003_2021_1899_MOESM3_ESM.pdf]

Description of Additional Supplementary Files

File name: Supplementary Data 1

Description: List of differentially expressed genes between Caco2 cells grown in flat/2D and organotypic/3D cultures.

File name: Supplementary Data 2

Description: List of metabolite GC/MS data as area under the curve (AUC) normalized to cell number.

File name: Supplementary Data 3

Description: The source data underlying the graphs and charts presented in the main figures
