# Supplementary material for: Architectural control of metabolic plasticity in epithelial cancer cells
Source: Commun Biol. 2021 Mar 19;4:371. doi: 10.1038/s42003-021-01899-4 (PMC7979883; doi:10.1038/s42003-021-01899-4)
Supplement: Supplementary file 7 — Reporting Summary [file 42003_2021_1899_MOESM7_ESM.pdf]

nature research

Corresponding author(s): Luke McCaffrey
Last updated by author(s): Jan 23, 2021

Reporting Summary

Nature Research wishes to improve the reproducibility of the work that we publish. This form provides structure for consistency and transparency in reporting. For further information on Nature Research policies, see our Editorial Policies and the Editorial Policy Checklist.

Statistics

For all statistical analyses, confirm that the following items are present in the figure legend, table legend, main text, or Methods section.

n/a	Confirmed	
☐	☒	The exact sample size (*n*) for each experimental group/condition, given as a discrete number and unit of measurement
☐	☒	A statement on whether measurements were taken from distinct samples or whether the same sample was measured repeatedly
☐	☒	The statistical test(s) used AND whether they are one- or two-sided<br>*Only common tests should be described solely by name; describe more complex techniques in the Methods section.*
☐	☒	A description of all covariates tested
☐	☒	A description of any assumptions or corrections, such as tests of normality and adjustment for multiple comparisons
☐	☒	A full description of the statistical parameters including central tendency (e.g. means) or other basic estimates (e.g. regression coefficient) AND variation (e.g. standard deviation) or associated estimates of uncertainty (e.g. confidence intervals)
☐	☒	For null hypothesis testing, the test statistic (e.g. *F*, *t*, *r*) with confidence intervals, effect sizes, degrees of freedom and *P* value noted<br>*Give P values as exact values whenever suitable.*
☒	☐	For Bayesian analysis, information on the choice of priors and Markov chain Monte Carlo settings
☒	☐	For hierarchical and complex designs, identification of the appropriate level for tests and full reporting of outcomes
☒	☐	Estimates of effect sizes (e.g. Cohen's *d*, Pearson's *r*), indicating how they were calculated

Our web collection on statistics for biologists contains articles on many of the points above.

Software and code

Policy information about availability of computer code

Data collection	Miscroscopy images were captured using ZEN software (Zeiss). Seahorse XF24 data was collected using Wave software (Seahorse Biosciences, v2.4). Raw GC/MS data was processed using MassHunter software (Agilent).
Data analysis	Statistical analysis was performed with GraphPad Prism and JMP14 and Excel (v16.34). Growth analysis was performed using MATLAB R2019a. Image analysis was preformed using FIJI/ImageJ v1.52t. Transcriptomic data was analyzed using R package limma.

For manuscripts utilizing custom algorithms or software that are central to the research but not yet described in published literature, software must be made available to editors and reviewers. We strongly encourage code deposition in a community repository (e.g. GitHub). See the Nature Research guidelines for submitting code & software for further information.

Data

Policy information about availability of data

All manuscripts must include a data availability statement. This statement should provide the following information, where applicable:

- Accession codes, unique identifiers, or web links for publicly available datasets
- A list of figures that have associated raw data
- A description of any restrictions on data availability

The authors declare that the data supporting the findings of this study are available within the paper and its supplementary information files. Data not included are available from the corresponding authors upon reasonable request.

Field-specific reporting

Please select the one below that is the best fit for your research. If you are not sure, read the appropriate sections before making your selection.

☒ Life sciences ☐ Behavioural & social sciences ☐ Ecological, evolutionary & environmental sciences

For a reference copy of the document with all sections, see nature.com/documents/nr-reporting-summary-flat.pdf

Life sciences study design

All studies must disclose on these points even when the disclosure is negative.

Sample size	No sample-size calculation was performed. 2-5 biological replicates were performed with multiple experimental replicates. The data was reproducible.
Data exclusions	No data were excluded.
Replication	All attempts at replication were successful.
Randomization	All experiments were cell or biochemical. Therefore, randomization was not relevant to our study.
Blinding	RNA-seq analysis was performed blind. Investigators were not blinded for other experiments. Data were analyzed using scripts applied uniformly to all samples to exclude potential bias from manual analysis.

Reporting for specific materials, systems and methods

We require information from authors about some types of materials, experimental systems and methods used in many studies. Here, indicate whether each material, system or method listed is relevant to your study. If you are not sure if a list item applies to your research, read the appropriate section before selecting a response.

Materials & experimental systems

n/a	Involved in the study	
☐	☒	Antibodies
☐	☒	Eukaryotic cell lines
☒	☐	Palaeontology and archaeology
☒	☐	Animals and other organisms
☒	☐	Human research participants
☒	☐	Clinical data
☒	☐	Dual use research of concern

Methods

n/a	Involved in the study	
☒	☐	ChIP-seq
☒	☐	Flow cytometry
☒	☐	MRI-based neuroimaging

Antibodies

Antibodies used	Rabbit anti-b-Catenin (Cell Signaling Technology, Cat# D10A8); Par6B (1:100, Santa Cruz, sc-67393), E-cadherin (1:250, BD Transduction, 610181); rabbit anti-FLAG (1:1000; Delta Biolabs Cat# DB125), and mouse anti-tubulin (1:5000; Sigma, clone DM1A)
Validation	From vendor website: Western blot analysis of extracts from control HeLa cells or HeLa cells with an apparent in-frame truncation mutation in the gene encoding β-Catenin (lane 2) using β-Catenin (D10A8). The change in β-Catenin molecular weight in the mutated HeLa cells is consistent with an in-frame deletion. The antibody has been validated by over-expression and genetic experiments (BenchSci).<br>The FLAG-antibody was confirmed by over-expression.<br>The tubulin antibody has been validated by over-expression and genetic experiments (BenchSci).<br>The Par6B antibody has be validated by knockdown in house.<br>The E-cadherin antibody has been validated by over-expression and genetic experiments (BenchSci).

Eukaryotic cell lines

Policy information about cell lines

Cell line source(s)	Caco2, MCF7, A549, MCF10A, and Caco2 cells were from ATCC. OV90 were a gift from Dr. Patricia Tonin
Authentication	Caco2, MCF7, and OV90 were authenticated by STR analysis.
Mycoplasma contamination	All cell lines were negative for mycoplasma.

Commonly misidentified lines<br>(See ICLAC register)	None.
